# Optimization of Process Parameters for Laser-Directed Energy Deposition Coatings of FeCoNi + 1%Y_2_O_3_ High-Entropy Alloy Based on Response Surface Methodology

**DOI:** 10.3390/ma18040883

**Published:** 2025-02-18

**Authors:** Danlin Shao, Xiaolin Bi, Minsheng Hong, Ruifeng Li

**Affiliations:** School of Materials Science and Engineering, Jiangsu University of Science and Technology, Zhenjiang 212100, China; 222241806703@stu.just.edu.cn (D.S.); just212100@yeah.net (M.H.)

**Keywords:** Y_2_O_3_-reinforced high-entropy alloys, laser-directed energy deposition, response surface methodology, process optimization, oxide dispersion strengthening

## Abstract

In order to achieve precise shaping control of FeCoNi + 1%Y_2_O_3_ laser-directed energy deposition (LDED) coatings and to reveal the influence of LDED process parameters on coating morphology, the response surface methodology (RSM) is employed in this study. The process parameters, including laser power, scanning speed, and powder feeding rate, are comprehensively considered, with the dilution rate, width-to-height ratio, and cladding area as evaluation criteria. A regression model is established to analyze both the individual and interactive effects of process parameters on forming quality. The findings indicate that the ideal process parameters are a laser power of 706.8 W, scanning speed of 646.2 mm/min, and powder feeding rate of 12 g/min. Experimental validation shows that the mean actual errors compared to the predicted values for dilution rate, width-to-height ratio, and cladding area are 7.36%, 10.03%, and 3.50%, respectively, proving the reliability of the model. The findings provide a theoretical basis for the prediction and control of the morphology of high-entropy alloy deposited layers with the addition of Y_2_O_3_.

## 1. Introduction

In 2004, Yeh et al. first proposed the concept of high-entropy alloys (HEAs) [1]. Alloys composed of five or more metal elements melted in equimolar or near-equimolar ratios exhibit microscopic structural characteristics, such as simplified microstructures, a reduced tendency to form intermetallic compounds, nanometer-sized precipitates, and amorphous structures. These alloys possess excellent properties, including high strength, high hardness, resistance to tempering, and wear resistance [2,3]. The components of HEAs are beneficial for achieving performance complementarity; however, excessive compositional complexity does not significantly enhance the alloy’s performance but rather increases manufacturing costs. Therefore, HEAs composed of Fe, Co, and Ni, which offer excellent performance at a lower cost, have gradually become the focus of research. The alloy composed of three ferromagnetic transition metals features high saturation magnetization, low coercivity, and high magnetic permeability, making them widely applicable in soft magnetic materials [4,5,6].

Y_2_O_3_, as a type of rare earth oxide, significantly improves the microhardness and mechanical properties of coatings [7,8,9]. In recent years, researchers have proposed adding rare earth elements (or rare earth oxides) to high-entropy alloy powder systems. The microalloying effect, grain boundary purification, grain boundary strengthening, and suppression of columnar crystal growth induced by rare earth elements can effectively enhance the microstructure and properties of the coating. Moreover, the addition of Y_2_O_3_ forms oxide-dispersion-strengthened (ODS) alloys, which exhibit exceptional performance [10]. ODS alloys maintain excellent high-temperature strength and oxidation resistance even at temperatures above 1000 °C, making them suitable for advanced applications such as aerospace engines, gas turbines, and other high-temperature oxidation-resistant components. Debta et al. [11] concluded that Y_2_O_3_ can refine the microstructure of coatings, enhance their fracture toughness and ductility, and reduce their wear rate. Fu et al. [12] discovered that when laser cladding iron-based alloy powders containing different amounts of La_2_O_3_ on rail materials, the addition of La_2_O_3_ decreased the width of the interface fusion zone, reduced the number of columnar crystals in the coating, and refined the grains. Cui et al. [13] successfully prepared FeCoNiCrMo HEA coatings and CeO_2_/FeCoNiCrMo composite coatings on the surface of TC4 alloy using laser cladding technology. The results showed that the addition of CeO_2_ powder reduced the crack sensitivity of the HEA coating, refined the grains, and improved the coating’s strength and toughness.

Laser-directed energy deposition (LDED) is an additive manufacturing technique that uses high-energy laser beams as the heat source [14]. The main parameters of the laser additive manufacturing process include scanning speed, laser power, powder feeding rate, defocus amount, and overlap rate [15]. Different LDED process parameters can affect the deposition efficiency, cracking, surface roughness, and morphology of high-entropy alloy deposits. Therefore, optimizing these parameters is of significant importance for improving the quality of additive manufacturing [16,17,18].

The response surface methodology (RSM) is a statistical technique that uses experimental design to obtain data and then fits a multivariate regression equation to describe the relationship between factors and responses [19]. By using RSM, an approximate model can be determined with a limited number of experimental data, guiding subsequent design or testing, and effectively saving experimental costs. Zhao Yong et al. [20] used RSM to study the influence of the input processing parameters, i.e., laser power, surface speed, carrier-gas flow rate, and string overlap (%), on the output responses, obtaining optimized processing parameters of T800 coatings without macroscopic cracks applied by EHLA. J. Deeying et al. [21] established a regression model for laser-soldering parameters and desired responses based on RSM to analyze the effect of the nitrogen gas pressure, wait time, laser energy, and focal position to the solder joints in Head Gimbal Assembly.

It is evident that LDED can provide a fast, low-cost, and high-precision shaping method for the fabrication of FeCoNi-Y_2_O_3_ alloys. The dilution rate, width-to-height ratio, and cladding area are important parameters for evaluating LDED forming quality, all of which are influenced by various process factors. The interactions between these factors are highly complex, and reasonable process parameters are key to ensuring the successful execution of LDED. This paper uses the response surface methodology (RSM) and Box–Behnken design (BBD) to establish mathematical regression models for the dilution rate, width-to-height ratio, and cladding area during LDED forming, optimizing the laser additive manufacturing process parameters for FeCoNi-Y_2_O_3_ alloys. Finally, secondary experiments are conducted using the optimized process parameters, and the reliability and accuracy of the multi-objective optimized parameter matching are verified through microscopic testing.

The optimized LDED process parameters offer significant benefits across multiple industries. In the aerospace sector, precise control of dilution rate and cladding morphology ensures high-performance protective coatings for turbine blades, combustion chambers, and other high-temperature components. These coatings enhance wear resistance and thermal stability, extending service life under extreme conditions. For the automotive industry, the optimized parameters enable efficient fabrication of wear-resistant coatings for engine pistons, transmission gears, and exhaust systems. This reduces friction losses and improves fuel efficiency while lowering maintenance costs. In the biomedical field, the ability to tailor the aspect ratio and cladding area with minimal errors supports the development of biocompatible coatings for orthopedic implants or surgical tools, enhancing corrosion resistance and mechanical durability in physiological environments.

The findings provide industries with a reliable framework to achieve reproducible, high-quality coatings. This reduces trial-and-error iterations in production, cuts material waste, and accelerates the adoption of LDED technology for customized industrial solutions.

## 2. Experimental Setup

### 2.1. Materials and Equipment

FeCoNi alloy powder and Y_2_O_3_ powder were weighed using an electronic balance. The powders were mixed in a mass ratio of 99:1 and placed in a ball mill for uniform mixing. The machine utilized for ball milling was a planetary ball mill operating in all directions at a rotation speed of 400 rpm for a mixing time of 6 h. The ball-to-powder ratio was 3:1. After ball milling, the powder underwent three hours at 180 °C in a dryer to guarantee drying and improve powder flow ability. The 316L stainless steel substrate, with dimensions of 100 mm × 10 mm × 10 mm (length × width × height), was used in the experiment. Prior to the experiment, the surface oxidation layer and oil contamination were removed from the substrate using an angle grinder, and it was cleaned with anhydrous ethanol.

**Figure 1:**
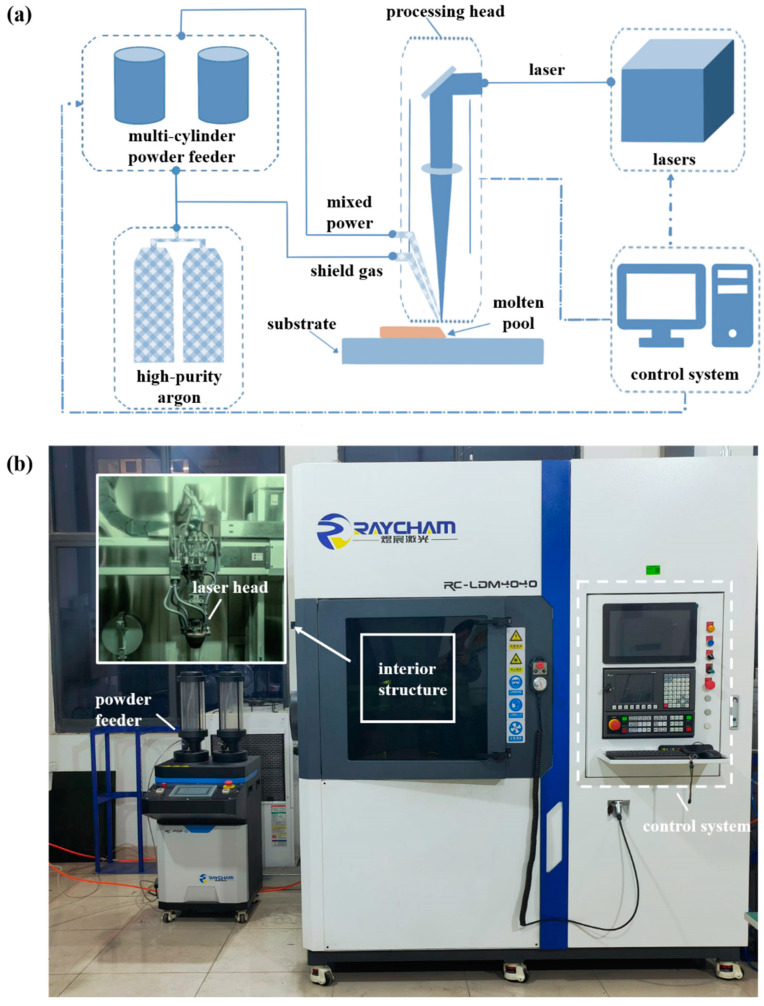
The LDED processing system: (**a**) schematic diagram; (**b**) picture.

The LDED forming equipment used was the RC-LDM4040 system produced by Nanjing Zhongke Yuchen Laser Technology Co., Ltd. (Nanjing, China). This system consists of several components, including a laser, water cooling unit, powder feeding system, electrical control cabinet, and CNC machine tools. The schematic diagram and picture of the forming system are shown in Figure 1.

### 2.2. Experimental Design and Methodology

**Figure 2:**
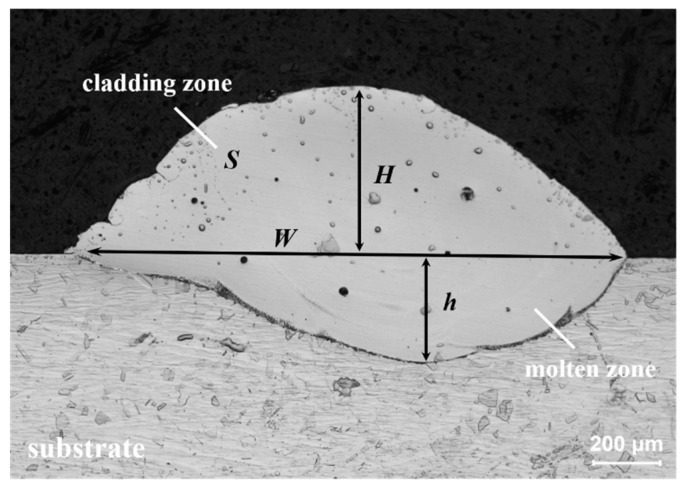
Cross-section characteristics of single LDED track morphology.

The laser-directed energy deposition (LDED) technique utilizes a single-track as the fundamental forming unit. Figure 2 illustrates a representative cross-sectional profile of the track, with essential dimensional parameters: melting height H, melting depth h, melting width W, and cladding zone S. The schematic of the single-track morphology outlines the dimensions required for the experimental output results. Equations (1) and (2) provide the definitions for the dilution rate D and width-to-height ratio α. The dilution rate (D) is a vital metric for assessing the metallurgical integration between the substrate and the deposited material. Reduced D values indicate restricted intermixing at the contact, which could compromise adhesion if excessively diminished [22]. The cladding layer morphology is significantly represented by the width-to-height ratio (α). The cladding zone to some extent reflects the deposition efficiency, and enhanced deposition efficiency is essential for industrialization. The cladding zone (S) further indicates process efficiency, as optimal energy distribution in this area improves both quality and throughput.

(1) $$D=\frac{h}{H+h}$$

(2) $$\alpha =\frac{W}{H}$$

Table 1 outlines the coded levels assigned to each experimental parameter. The Box–Behnken design (BBD) approach enables an efficient analysis of three factors at three levels, reducing the number of experimental runs required to assess nonlinear factor interactions across multiple variables. This eliminates the need for extensive sequential testing, making BBD a practical tool for parameter optimization in manufacturing processes [23,24]. Using the BBD design method, an accurate model can be established to describe the relationship between process parameters and their interactions with response values. The methodology relies on multivariate regression analysis to formulate and analyze empirical relationships between inputs and outputs. For this investigation, the interplay between parameters and response metrics is quantified through polynomial Equation (3) [25].

(3) $$y=\beta_{0}+\sum_{j=1}^{k}{\beta_{j}\chi }_{j}+\sum_{i,j=1}^{k}{\beta_{ij}\chi }_{i}\chi_{j}+\sum_{j=1}^{k}\beta_{jj}\chi_{j}^{2}+\varepsilon$$

In the formula, y represents the response value, β_0_ is the intercept coefficient, and β_j_, β_ij_, and β_jj_ are the regression coefficients for the linear, interaction, and quadratic terms, respectively. χ_j_ represents the process parameters, k is the number of factors, and ε is the residual error.

## 3. Results and Analysis

### 3.1. Experimental Results

Based on the three-factor, three-level relationships shown in Table 1, the BBD method requires a total of 15 single-track deposition trials. After forming the single-track deposition layers, they were subjected to wire cutting, embedding, grinding, polishing, and etching. The cross-sectional morphology of the single track was observed using a Zeiss optical microscope. The melting width, melting height, and melting depth were measured, and the dilution rate and width-to-height ratio were calculated using Equations (1) and (2). The cladding area SSS was determined using ImageJ 2024 software. The experimental plan and results are shown in Table 2.

Experimental data were processed through Design-Expert 13 software, and a second-order regression model was formulated to characterize the correlation between morphological characteristics and individual processing parameters.

### 3.2. Dilution Rate Mathematical Model and Analysis of Influencing Factors

Analysis of variance (ANOVA) is used to analyze the significance of each factor by determining the proportion of total variation caused by the variation of independent variables. The F-value is an important indicator for determining the significance of the model; the larger the F-value, the more significant the impact of that factor on the model. The *p*-value indicates the degree to which the F-value can be rejected; a smaller *p*-value means a higher level of confidence in the F-value [26]. At the same time, the lack-of-fit test aims to determine whether any important variables are missing from the model. The model is considered to have a high degree of fit only when the lack-of-fit term is not significant [27]. The coefficient of determination R^2^ is used to assess the degree to which the independent variables explain the response variable, calculated as the ratio of the regression sum of squares to the total sum of squares. The larger the R^2^, the more accurate the model’s prediction [28]. To assess whether overfitting exists in the regression model, the difference between the predicted coefficient of determination R_pre_^2^ and the adjusted coefficient of determination R_adj_^2^ can be used. A smaller difference indicates better model fitting.

ANOVA was conducted on the experimental results, and the mathematical model for dilution rate was obtained, as shown in Equation (4), with the fitting variance summarized in Table 3. Here, A represents laser power, B represents scanning speed, and C represents powder feed rate. The ANOVA for the dilution rate model showed that the *p*-value was less than 0.0001, and the lack-of-fit *p*-value was greater than 0.05, indicating that the model had a high degree of fitting accuracy. The coefficient of determination R^2^ was 0.8731, meaning that the predicted values were very close to the actual values. The adjusted R_adj_^2^ was very close to R^2^, indicating that there were no redundant coefficients in the model. The coefficient of variation (CV) was 8.95%, well below the 10% threshold commonly recognized in material science, indicating excellent experimental reproducibility. This value falls within a reasonable and widely accepted range, further confirming the reliability of the model and its potential to effectively guide and predict subsequent experimental outcomes [29,30,31].

(4) D=40.68+6.55×A+3.02×B−8.56×C

**Figure 3:**
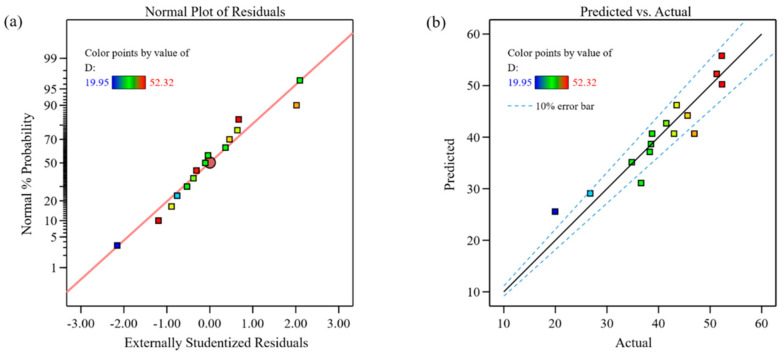
Diagnostic plots for the dilution rate model: (**a**) normal plot of residuals; (**b**) comparison of predicted and actual values.

Figure 3 shows the diagnostic plots for the dilution rate model. Figure 3a presents the probability distribution of the residuals, where all the scatter points are closely aligned along a straight line, indicating that the residuals approximately follow a normal distribution. Figure 3b analyzes the agreement between the predicted and actual values, where the y = x line represents perfect prediction. The dashed lines denote the ±10% symmetric error bars, which define the acceptable range of relative error for model validation. Most data points fall within this error band, indicating that the model predictions are consistent with the experimental measurements within the 10% tolerance threshold. This level of accuracy aligns with the standards adopted in each field, where errors below 10% are deemed acceptable for practical applications. The few outliers beyond the 10% range may be because the parameters exhibit nonlinear effects (such as interaction terms or quadratic terms), and the model only considers linear terms.

**Figure 4:**
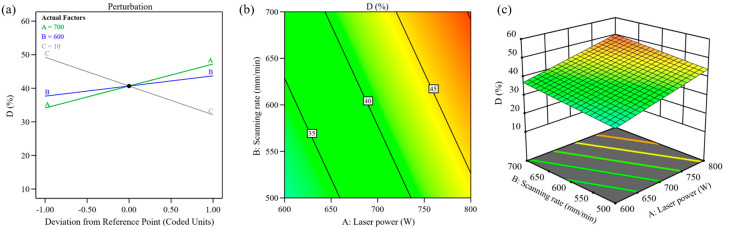
Relationship between laser processing parameters and cladding layer dilution rate: (**a**) perturbation; (**b**) contour; (**c**) 3D surface.

A higher material feed rate inversely impacts dilution. Increased powder delivery absorbs more laser energy, boosting clad height but limiting energy transfer to the substrate. This reduces melt depth and lowers dilution, aligning with theoretical predictions. The influence of process parameters on dilution rate is shown in Figure 4. From Figure 4a, it can be seen that laser power, scanning speed, and powder feeding rate are all significant factors affecting the dilution rate of the cladding layer. Elevated laser power correlates with higher dilution, as greater thermal input promotes substrate melting. Similarly, faster scanning speeds amplify dilution due to the reduced energy per-unit area and lower material deposition per-unit time. These conditions diminish cladding height while directing more laser energy into the substrate, deepening the molten pool, and raising dilution. The dilution rate demonstrates an inverse linear relationship with the powder feeding rate. Higher powder feeding rates correspond to lower dilution rates. This phenomenon occurs because elevated powder input introduces more material into the laser beam per-unit time, increasing energy absorption. As a result, the cladding layer increases in thickness, leading to a decrease in the energy transferred to the substrate and a reduction in penetration depth into the base material. As described by the dilution rate equation, these combined effects lower the overall dilution rate. Experimental data in Figure 4b,c reveal that at a 10 g/min powder feeding rate, combining moderate scanning speeds with reduced laser power achieves minimal dilution. This balance optimizes energy distribution, ensuring sufficient cladding formation while limiting substrate interaction. The findings align with theoretical expectations, confirming that controlled process parameters effectively manage dilution in laser-based material deposition applications.

### 3.3. Mathematical Model of the Width-to-Height Ratio and Analysis of Its Influencing Factors

ANOVA findings for the width-to-height ratio reveal that the chosen model yields a *p*-value below 0.0001, while the lack-of-fit *p*-value exceeds 0.05, confirming the model’s reliability in fitting the experimental data. The model’s signal-to-noise ratio (Adeq Precision) reaches 23.6174, substantially surpassing the threshold of 4, thereby validating its predictive capability. Additionally, the model’s R^2^ is 0.9322. A comparison between adjusted R^2^ (0.9138) and predicted R^2^ (0.8733) shows a marginal discrepancy of 0.0405, well under the acceptable limit of 0.2, indicating that the model can accurately predict the error in the experimental values. By comparing the *p*-values of the input variables, it can be concluded that scanning speed and powder feeding rate are significant factors affecting the width-to-height ratio. The model relationship is given in Equation (5), and the fitting variance is shown in Table 4.

(5) α=3.75+0.0275×A+0.5587×B−0.7238×C

**Figure 5:**
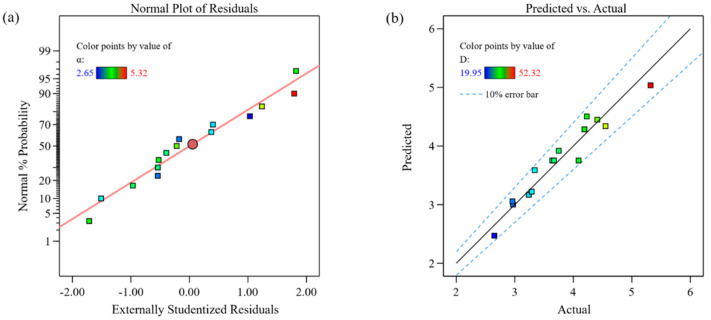
Width-to-height ratio model indicator plot: (**a**) normal plot of residuals; (**b**) comparison of predicted and actual values.

Figure 5a shows the normal probability plot of the standardized residuals of the regression model. The scattered points are distributed along a straight line, indicating that the residuals follow a normal distribution. The comparison between the actual and predicted values is shown in Figure 5b, where the scattered points are distributed near the straight line, indicating that the experimental values are close to the model’s predicted values, further confirming the validity of the constructed model.

**Figure 6:**
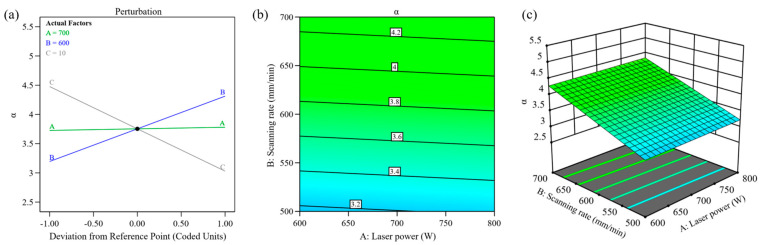
Relationship between laser processing parameters and the width-to-height ratio of the cladding layer: (**a**) perturbation; (**b**) contour; (**c**) 3D surface.

From Figure 6a, it can be observed that the width-to-height ratio is positively correlated with scanning speed and negatively correlated with powder feed rate, while the influence of laser power on the width-to-height ratio is not significant. Figure 6b,c show the interaction plots of the width-to-height ratio with laser power and scanning speed (at a powder feed rate of 10 g/min). The change in slope caused by scanning speed variation is slightly greater than that caused by laser power variation, indicating that scanning speed has a larger impact than laser power, which is consistent with the previous *p*-value analysis results. When laser power is fixed, the width-to-height ratio of the cladding layer increases with the increase in scanning speed. The variation curve of the width-to-height ratio with laser power is relatively flat, suggesting that laser power has a small influence on the width-to-height ratio of the cladding layer. The shape of the contour lines reflects the strength of the interaction; when the contour lines are elliptical or saddle-shaped, it indicates a significant interaction between the two factors [32]. From the contour plot in Figure 6b, it can be seen that the contour lines are neither elliptical nor saddle-shaped, indicating that the interaction between laser power and scanning speed on the width-to-height ratio is not significant. From bottom to top, the width-to-height ratio of the cladding layer gradually increases. Therefore, to achieve a larger width-to-height ratio of the cladding layer, laser power should be moderate, and a larger scanning speed should be selected.

### 3.4. Cladding Area Variance and Model Analysis

The experimental data underwent quadratic regression analysis to establish a mathematical model characterizing the cladding area, as presented in Equation (6). Variance analysis for the fit is detailed in Table 5. Among these, A·B represents the interaction between scanning speed and laser power, and A^2^ is the quadratic term for laser power, with other terms having the same meanings as previously explained. According to Table 5, the cladding area model exhibits a *p*-value below 0.0001, and the lack-of-fit *p*-value exceeds 0.05, confirming the model’s statistical significance and adequate alignment with the data. An R^2^ value of 0.9946 suggests that the model’s predictions align closely with observed data. Furthermore, adjusted R^2^ (0.9848) and predicted R^2^ (0.9254) values differ by less than 0.2, implying no overfitting in the regression. The signal-to-noise ratio is 32.4580, which is greater than 4, indicating a high degree of model fitting and the ability to obtain a relatively accurate mathematical model. Additionally, the coefficient of variation (CV) of 3.57% falls within acceptable limits, further validating the model’s consistency and dependability under experimental conditions. By comparing the *p*-values of the input variables, it is observed that the first-order terms A, B, and C significantly affect the cladding area, while the interaction terms AC, BC, along with the quadratic terms B^2^ and C^2^ have notable effects. Other factors do not show significant influence. Based on the mean square values, the influence of the process parameters on the cladding area is ranked as C > B > A.

(6) $$S=0.5087+0.0947\times A-0.1262\times B+0.1373\times C-0.0167\times AB\;+0.0296\times AC-0.0342\times BC+0.0180\times A^{2}+0.0349{\times B}^{2}+0.0502\times C^{2}$$

**Figure 7:**
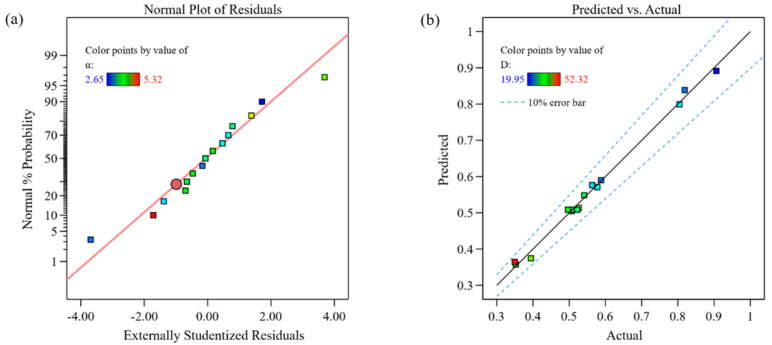
Cladding area model indicators. (**a**) normal plot of residuals; (**b**) comparison of predicted and actual values.

**Figure 8:**
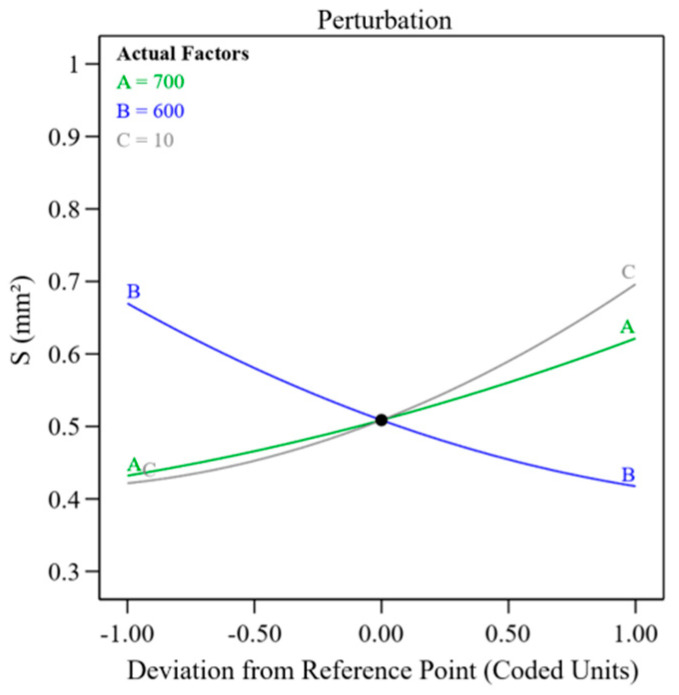
Perturbation analysis of process parameters on cladding area.

Figure 7a shows the residual distribution of the cladding area model. The points are consistently aligned along a straight line, indicating that the model has good stability. Figure 7b compares the predicted values with the actual values, where both the predicted and experimental values are almost aligned along a 45° line, confirming that the established model for the relationship between process parameters and the optimized target cladding area is highly reliable. Figure 8 presents the sensitivity analysis of each factor on the cladding area. It can be seen that the cladding area is positively correlated with laser power and powder feed rate, and negatively correlated with scanning speed.

**Figure 9:**
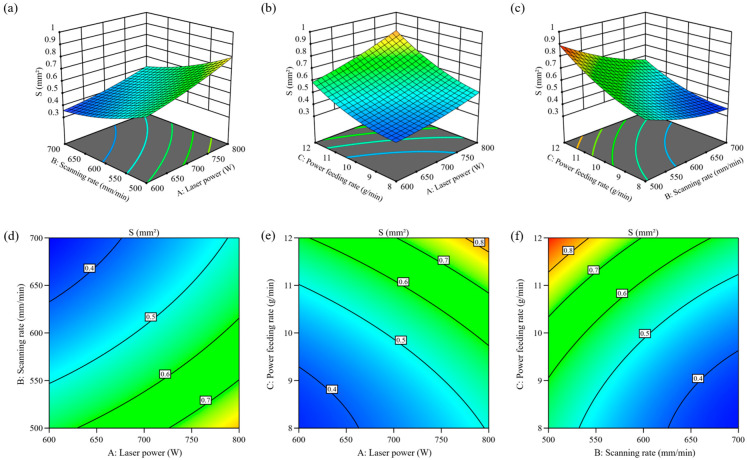
Response surface and contour plots of cladding area. (**a**,**d**) Scanning Speed and Laser Power; (**b**,**e**) Laser Power and Powder Feeding Rate; (**c**,**f**) Scanning Speed and Powder Feeding Rate.

As shown in Figure 9, the three-dimensional response surface model visually demonstrates parameter interactions. Analysis reveals that maintaining fixed laser power while elevating scanning velocity shortens the duration of laser–material interaction, consequently diminishing the molten zone’s liquid-phase volume. Meanwhile, a decrease in the powder feeding rate results in a reduced powder supply in the molten pool, ultimately leading to a smaller cladding area. When stabilizing scanning velocity, heightened laser power amplifies energy delivery per-unit time to the molten pool, enlarging its liquid-phase domain. Additionally, raising the powder supply rate enhances material input, further promoting the increase of the cladding area. Under consistent powder delivery conditions, enhanced laser power similarly elevates thermal energy input per-unit time, expanding the molten zone’s fluid state region. Notably, even with the prolonged laser interaction time caused by the reduced scanning velocity, larger cladding areas were achieved under these conditions. This may be because the reduction in scanning speed allows the laser energy to distribute more evenly within the molten pool, thereby facilitating the deposition process.

## 4. Model Validation and Process Parameter Optimization

### 4.1. Multi-Objective Optimization for Optimal Solution

The dilution rate serves as a critical parameter for assessing the effectiveness of the deposited layer. Elevated dilution levels may cause an overly extensive heat-affected region in the substrate, raising the risk of crack formation and porosity, which compromises layer integrity and functionality. A greater width-to-height ratio reflects enhanced adhesion between the high-entropy alloy and substrate. Similarly, a larger cladding area corresponds to improved deposition efficiency. Thus, process optimization aims to reduce dilution rates while simultaneously increasing both the width-to-height ratio and cladding dimensions. The importance ranking of the three parameters is: dilution rate > cladding area > width-to-height ratio.

Based on the regression models for dilution rate, width-to-height ratio, and cladding area, the “in range” target range was set using Design-Expert software. The target ranges are as follows: dilution rate from 19.95% to 52.32%, width-to-height ratio from 2.65 to 5.32, and cladding area from 0.349 to 0.906 mm^2^. The constraint ranges for the laser power, scanning speed, and powder feeding rate are 600–800 W, 500–700 mm/min, and 8–12 g/min, respectively. The optimized laser process parameters obtained are: laser power 706.8 W, scanning speed 646.2 mm/min, and powder feeding rate 12 g/min.

### 4.2. Experimental Verification

Based on the above-optimized parameters, a single-track deposition experiment involving FeCoNi-Y_2_O_3_ high-entropy alloy (HEA) was performed via laser-directed energy deposition (LDED). The outcomes are presented in Table 6. Theoretical estimates for dilution rate, width-to-height ratio, and cladding area were computed using Equations (4)–(6), whereas experimental measurements were derived through Equations (1) and (2) and Image J software. To compare the experimental and predicted accuracy, the error Δ is defined as:

(7) $$\Delta =\frac{\begin{array}{c} \left|x_{actual}-x_{predicted}\right| \end{array}}{x_{predicted}}\times 100\%$$

The corresponding prediction errors are shown in Table 6. Data analysis reveals average deviations of 7.36% for dilution rate, 10.03% for width-to-height ratio, and 3.50% for cladding area. The discrepancies between the actual measurements and theoretical values can be attributed to factors such as the neglect of insignificant influencing factors during modeling, irregularities in the actual molten pool, and fluctuations in the actual output parameters of the equipment. The most common cause is manual measurement errors when quantifying cladding areas and lengths using ImageJ software, especially for coatings with complex geometries. Overall, the mean errors are within an acceptable range, confirming the model’s predictive accuracy.

### 4.3. Microstructure and Properties of the Optimized Cladding Sample

**Figure 10:**
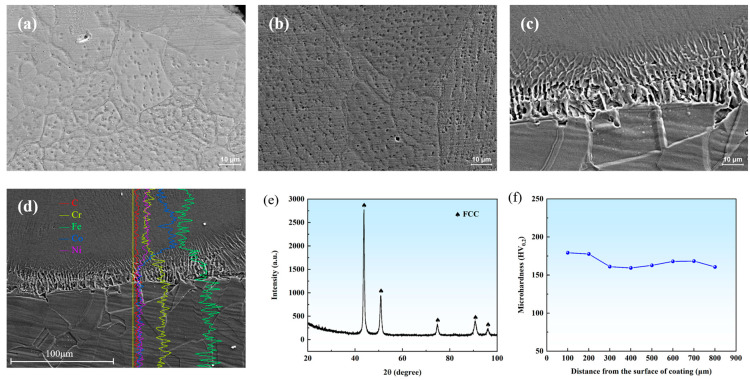
SEM images of coatings from (**a**) the top region, (**b**) central region, (**c**) bottom region, (**d**) EDS line scanning results from the substrate to the coating. (**e**) XRD result of coating. (**f**) microhardness of coating.

Based on the above LDED parameter optimization, LDED experiments were conducted using a laser power of 706.8 W, a scanning speed of 646.2 mm/min, and a powder feeding rate of 12 g/min to obtain FeCoNi + 1% Y_2_O_3_ coatings. Figure 10a–c illustrate the SEM microstructure of the deposited layer in the upper, middle, and lower areas. Columnar development and an increase in grain size are seen as deposition progresses from the bottom to the top. Figure 10d depicts the elemental distribution at the coating–substrate interface, indicating increased iron levels and diminished cobalt concentrations at this juncture, which imply interdiffusion and robust metallurgical bonding. Figure 10e presents the XRD diffraction results of the cladding layer, where only the FCC phase is formed in the cladding layer. Figure 10f shows the microhardness profile of the cladding layer along its depth, with an average hardness of approximately 167 HV.

## 5. Discussion

This study employs response surface methodology (RSM) to optimize laser-directed energy deposition (LDED) parameters for FeCoNi + 1% Y_2_O_3_ coatings, focusing on dilution rate, width-to-height ratio, and cladding area as key evaluation criteria. This approach aligns with recent trends in additive manufacturing research, where RSM has been widely used to address multi-parameter interactions and achieve optimal solutions. For instance, Chenet al. [33] utilized RSM combined with NSGA-II algorithms to optimize laser-deposited high-entropy alloys (HEAs), while Dong et al. [25] applied RSM and multi-objective genetic algorithms to AlCoCrFeNi_2_._1_ eutectic HEA coatings, prioritizing hardness and wear resistance. Furthermore, the study demonstrates high validation accuracy, with mean errors between predicted and experimental results being 7.36% for dilution rate, 10.03% for width-to-height ratio, and 3.50% for cladding area. This level of reliability surpasses that of hybrid RSM-GA models in studies like Dill et al. [34], where deviations in surface morphology predictions exceeded 15%.

A significant innovation in this work is the incorporation of 1% Y_2_O_3_ into FeCoNi HEAs, which introduces distinct advantages over conventional HEAs. While prior studies, such as those on CoCrFeNi coatings by Chenet al. [33], focused on grain refinement through process parameter tuning, they often overlooked the potential of oxide dispersion strengthening. In contrast, the addition of Y_2_O_3_ in this study likely enhances thermal stability and refines the microstructure, as observed in other oxide-reinforced systems like Al_2_O_3_ in Ni-based coatings. Y_2_O_3_ acts as both a grain refiner and an oxygen scavenger, mitigating defects such as porosity and improving interfacial bonding.

The economic viability and scalability of the optimized LDED process are essential for its industrial adoption. Compared to conventional trial-and-error approaches, the statistical optimization framework developed in this study significantly reduces material and operational costs. The predictive accuracy of the response surface model minimizes the need for iterative experiments, saving approximately 20–30% in time and resources during process development. Furthermore, the optimized parameters can enhance deposition efficiency. The high efficiency, combined with moderate energy consumption, positions the process as a cost-effective solution for large-scale production. For example, in automotive applications, scaling up this method could lower the per-unit cost of wear-resistant engine components by reducing material waste and energy use, while maintaining consistent coating quality across batches.

Regarding environmental impact, the LDED process inherently offers advantages over traditional subtractive manufacturing methods by minimizing material wastage. Our optimized parameters further enhance sustainability through improved powder utilization rates and reduced post-processing requirements. However, we acknowledge that the energy consumption of LDED remains a concern, particularly in industries prioritizing carbon neutrality. To address this, future work could explore hybrid manufacturing strategies—such as combining LDED with renewable energy sources or recycling waste heat—to improve overall energy.

In the future, the research will focus on leveraging the existing dataset to refine the optimization model and enhance the reliability of the regression analysis. This will involve conducting residual analysis to identify and address any deviations between predicted and experimental values, ensuring the model’s accuracy. Additionally, checks for multicollinearity among process parameters will be performed to eliminate redundant variables that could distort the results. Outlier detection techniques will also be employed to identify and mitigate data points that may skew the model’s predictions. These steps will collectively strengthen the robustness of the regression model, providing a more reliable foundation for process optimization. Building on the optimized parameters, the next phase will also involve a comprehensive evaluation of the mechanical properties of the FeCoNi + 1% Y_2_O_3_ coatings. This will include testing for toughness, wear resistance, bonding strength, and tribological performance.

Taken together, this study advances LDED research by systematically linking Y_2_O_3_-reinforced HEA coatings to process parameter optimization via RSM. Future efforts could explore the integration of advanced machine learning algorithms to bridge gaps in large-scale industrial applicability.

## 6. Conclusions

This study investigates the influence of laser power, scanning speed, and powder feeding rate on the macro morphology of medium-entropy alloy deposition layers during the laser deposition process using the response surface method (RSM) and the BBD design. Mathematical models delineating the link among processing parameters, dilution rate, width-to-height ratio, and cladding area are formulated, and optimal process parameters are derived using multi-objective optimization. The main conclusions are as follows:

(1) Dilution rate is most strongly influenced by powder feed rate, with laser power and scanning speed showing comparatively smaller impacts. Width-to-height ratio is primarily affected by powder feed rate and scanning speed, whereas laser power contributes minimally. All three parameters—laser power, scanning speed, and powder feed rate—demonstrate substantial effects on cladding area, with each variable playing a critical role in its formation.

(2) Dilution rate exhibits a proportional relationship with laser power and scanning speed but inversely correlates with powder feeding rate. For width-to-height ratio, scanning speed shows a positive association, while powder feeding rate has a negative impact, and laser power demonstrates minimal influence. The cladding area exhibits a positive correlation with laser power and powder feeding rate, while demonstrating a negative correlation with scanning speed.

(3) Mathematical models for the dilution rate, width-to-height ratio, and cladding area in relation to laser power, scanning speed, and powder feeding rate were established based on the response surface method. The optimized parameters identified through this analysis include a laser power of 706.8 W, a scanning speed of 646.2 mm/min, and a powder feeding rate of 12 g/min. Average prediction deviations for dilution rate, width-to-height ratio, and cladding area measured 7.36%, 10.03%, and 3.50%, respectively, validating the model’s predictive accuracy.

## Figures and Tables

**Table 1:** Level coding table of each process parameter.

Process Parameters	Unit	Symbol	Coding Level		
			−1	0	1
Laser power	W	A	600	700	800
Scanning rate	mm/min	B	500	600	700
Powder feeding rate	g/min	C	8	10	12

**Table 2:** Experimental design and data.

Test Number	Level of Process Parameters			Value of Morphological Feature		
	A (W)	B (mm/min)	C (g/min)	D (%)	α	S (mm^2^)
1#	1	−1	0	45.63	3.29	0.8044
2#	0	−1	1	26.76	2.65	0.9061
3#	−1	1	0	38.3	4.19	0.3523
4#	−1	−1	0	36.62	3.24	0.5635
5#	−1	0	−1	41.5	4.41	0.3943
6#	0	1	−1	51.29	5.32	0.3498
7#	1	0	−1	52.26	4.23	0.5065
8#	1	1	0	52.32	4.55	0.5263
9#	0	0	0	38.75	3.64	0.5221
10#	0	0	0	43	3.67	0.5074
11#	0	0	0	46.95	4.09	0.4965
12#	1	0	1	38.53	2.96	0.8188
13#	−1	0	1	19.95	2.97	0.5880
14#	0	−1	−1	43.51	3.75	0.5415
15#	0	1	1	34.81	3.34	0.5778

A—Laser power; B—Scanning rate; C—Powder feeding rate; D—dilution rate; α—width-to-height ratio; S—cladding area.

**Table 3:** Analysis of variance (ANOVA) for the dilution rate fitting model.

Source	Sum of Squares	Mean Square	F-Value	*p*-Value	Significance
Model	1002.73	334.24	25.23	<0.0001	Significant
A	342.83	342.83	25.87	0.0004	-
B	73.20	73.20	5.52	0.0385	-
C	586.70	586.70	44.28	<0.0001	-
Residual	145.75	13.25	-	-	-
Lack of Fit	112.12	12.46	0.7408	0.6929	Not significant
Pure Error	33.64	16.82	-	-	-
Cor Total	1148.49	-	-	-	-

A—Laser power; B—Scanning rate; C—Powder feeding rate.

**Table 4:** Variance analysis table for the width-to-height ratio fitting model.

Source	Sum of Squares	Mean Square	F-Value	*p*-Value	Significance
Model	6.99	2.23	50.45	<0.0001	Significant
A	0.0061	0.0061	0.1368	0.7185	-
B	2.50	2.50	56.47	<0.0001	-
C	4.19	4.19	94.74	<0.0001	-
Residual	0.4866	0.0442	-	-	-
Lack of Fit	0.3600	0.0400	0.6318	0.7424	Not significant
Pure Error	0.1266	0.0633	-	-	-
Cor Total	7.18	-	-	-	-

A—Laser power; B—Scanning rate; C—Powder feeding rate.

**Table 5:** Variance analysis table for cladding area model.

Source	Sum of Squares	Mean Square	F-Value	*p*-Value	Significance
Model	0.3727	0.0414	102.00	<0.0001	Significant
A	0.0718	0.0718	176.80	<0.0001	-
B	0.1273	0.1273	313.58	<0.0001	-
C	0.1509	0.1509	371.61	<0.0001	-
A·B	0.0011	0.0011	2.76	0.1578	-
A·C	0.0035	0.0035	8.65	0.0322	-
B·C	0.0047	0.0047	11.51	0.0194	-
A^2^	0.0012	0.0012	2.96	0.1462	-
B^2^	0.0045	0.0045	11.09	0.0208	-
C^2^	0.0093	0.0093	22.93	0.0049	-
Residual	0.0020	0.0004	-	-	-
Lack of Fit	0.0017	0.0006	3.43	0.2337	Not significant
Pure Error	0.0003	0.0002	-	-	-
Cor Total	0.3748	-	-	-	-

A—Laser power; B—Scanning rate; C—Powder feeding rate.

**Table 6:** Comparison of predicted and actual values under optimized parameters (laser power 706.8 W, scanning speed 646.2 mm/min, and powder feeding rate 12 g/min).

Experiment		Predicted Value	Actual Value	Δ/%
No.1	D	33.98	37.36	9.95
	α	3.29	2.84	13.68
	S	0.638	0.7062	10.69
No.2	D	33.98	33.34	1.88
	α	3.29	2.79	15.20
	S	0.638	0.7559	18.47
No.3	D	33.98	38.75	14.04
	α	3.29	3.26	0.91
	S	0.638	0.5189	18.67
Mean	D	33.98	36.48	7.36
	α	3.29	2.96	10.03
	S	0.638	0.6603	3.50

D—dilution rate; α—width-to-height ratio; S—cladding area.

## Data Availability

The original contributions presented in this study are included in the article. Further inquiries can be directed to the corresponding authors.
